# Silver Nanoclusters with Specific Ion Recognition Modulated by Ligand Passivation toward Fluorimetric and Colorimetric Copper Analysis and Biological Imaging

**DOI:** 10.1038/srep20553

**Published:** 2016-02-05

**Authors:** Zongzhao Sun, Shuying Li, Yao Jiang, Yuchun Qiao, Liyan Zhang, Lulu Xu, Jinghui Liu, Wei Qi, Hua Wang

**Affiliations:** 1 Shandong Province Key Laboratory of Life-Organic Analysis, College of Chemistry and Chemical Engineering, Qufu Normal University, Qufu, 273165, P. R. China; 2 Jining Functional Materials and Surface Treatment Technology R&D Center, Southern Shandong Academy of Engineering Technology, Jining City, Shandong Province 272000, P. R. China

## Abstract

Silver nanoclusters were synthesized and passivated by glutathione (GSH) ligand, with high aqueous stability and powerful red fluorescence and UV-vis yellow colour. Importantly, the specific recognition of the AgNCs was modulated from Hg^2+^ ions to Cu^2+^ ions upon the GSH passivation, of which the unique GSH-Cu^2+^ chelating reaction could conduct the fluorescence quenching of AgNCs. Strong UV-vis absorbance of GSH-passivated AgNCs could also be realized depending on the Cu^2+^ levels. Moreover, the Cu^2+^-induced loss of fluorescence and UV-vis absorbance of GSH-passivated AgNCs could be well restored by using stronger Cu^2+^ chelating agent. A simultaneous and reversible fluorimetric and colorimetric sensing method was thereby developed for probing Cu^2+^ ions in blood with high sensitivity and selectivity. Subsequently, the fluorescence-trackable imaging for live tissues and cells was demonstrated towards the analysis Cu^2+^ ions using GSH-passivated AgNCs as the fluorescent probes. This study indicates that the use of functional ligands like GSH could not only modulate the specific ion recognition of AgNCs, but also endow them the high aqueous stability and powerful red fluorescence towards the wide applications for ion sensing and biological imaging in the complicated media like blood.

In recent years, metal nanoclusters (NCs) with some distinct optical and catalytic properties have obtained increasing applications in the fields of chemical sensing, molecular labelling, biological imaging, and catalysis^1^,^2^,^3^,^4^. In particular, a variety of noble metal NCs, most known as AuNCs, AgNCs, and their alloy NCs, have been applied for detecting some toxic metal ions of great importance^5^,^6^,^7^,^8^,^9^. Moreover, many synthesis methodologies have been developed for preparing these unique fluorescent materials by using different organic or biological templates such as proteins, peptides, polyelectrolyte, and DNAs^5^,^10^,^11^. For example, luminescent AuNCs and Au@AgNCs were prepared in the protein matrix by the sonochemistry route for probing copper ions in water^5^. Polyelectrolyte was employed as the template to synthesize highly fluorescent AgNCs for the detection of Hg^2+^ and Cu^2+^ ions^10^. DNAs were also reported as the stabilizer for fabricating AgNCs to probe Cu^2+^ ions^11^. In these studies, these templates have only been recognized as the stabilizers in the synthesis of the fluorescent species, with the limited functional diversifications like the specific ion recognition. Particularly, how they could play the role in modulating the specific recognitions or responses of noble metal NCs to the meaningful metal ions have hardly been explored systematically. Therefore, the synthesis of metal nanoclusters with the specific recognition modulated by ligand passivation is an attractive but challenging target to pursue.

As the hazardous heavy metal ions in environment, copper ions may bring deleterious effects with too high concentrations in tissues. For example, the long-term exposure to copper ions of high levels can lead to cellular toxicity and liver or kidneys damage^12^,^13^. So far, many modern detection methods have been applied for targeting copper ions such as electrochemical detection, fluorescence analysis, and colorimetric assay^5^,^14^,^15^. For example, AgNCs have been widely documented for the fluorescent analysis of Cu^2+^ ions and/or Hg^2+^ ions, which might, however, be trapped by the interferences from co-existing metal ions that may challenge the specific ion detections^16^,^17^. Also, most of these methods might encounter with the low detection sensitivity and poor probing abilities against the background interferences. Therefore, it is of great interest to develop a simple, rapid, and highly sensitive detection method to explore copper ions in some complicated media especially in human body fluids (i.e., blood).

Glutathione (GSH), a ubiquitous antioxidant in human and plant cells, is a tri-peptide consisting of glutamic acid, cysteine, and glycine units. It possesses the reactive thiol groups with good affinity to metal ions, and enjoys amine and carboxylate groups for coupling with other molecules or ions of great interest^18^. As a result, GSH has been applied as an effective stabilizer in the synthesis of some metal NCs like AgNCs^19^ and AuNCs^20^. Also, GSH was demonstrated to possess the high affinity or chelating ability to Cu^2+^ ions^21^. In the present work, GSH was chosen alternatively as an example of passivation ligands to work with dihydrolipoic acid (DHLA) to synthesize water-soluble GSH-passivated AgNCs with considerably strong red fluorescence and yellow colour. Importantly, the specific ion recognition of AgNCs could be thus modulated to Cu^2+^ ions upon the GSH passivation, in contrast to the general AgNCs with response to Hg^2+^ ions^7^. Comparing to most of the detection methods documented for Cu^2+^ ions, the so developed detection method could achieve the selective analysis for Cu^2+^ ions in the mixtures co-existing other metal ions, i.e., Hg^2+^ ions. Particularly, the reversible fluorometric and colorimetric sensing assays with GSH-passivated AgNCs could be expected for copper ions. Subsequently, the application feasibility of GSH-passivated AgNCs for the fluorescence-trackable imaging of live cells and tissues was demonstrated towards the analysis of Cu^2+^ ions in the complicated media like blood.

## Results

**Figure 1:**
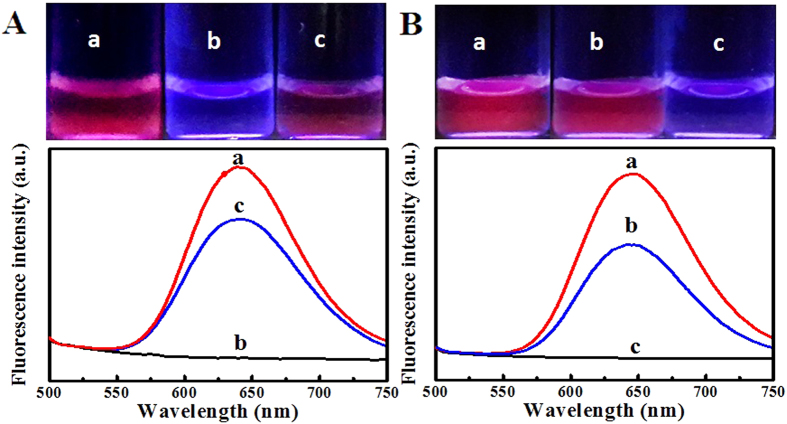
Comparison of the fluorescent responses to Hg^2+^ ions or Cu^2+^ ions between (**A**) AgNCs and (**B**) GSH-passivated AgNCs (5.0 μM) in the (a) absence and presence of (b) 1.0 μM Hg^2+^ ions or (c) Cu^2+^ ions, with the corresponding photographs of the testing solutions (top).

AgNCs were firstly synthesized and passivated with GSH in the presence of DHLA. The resulting GSH-passivated AgNCs were then characterized by the FT-IR spectra (Figure S1), taking GSH and DHLA as the controls. As shown in Figure S1, GSH-passivated AgNCs could present clearly the 1574.1 cm^−1^ band of carboxyl groups (from DHLA or GSH) and the 3496.3 cm^−1^ band of amine groups (from GSH), thus confirming the presence of DHLA and GSH. Furthermore, the S-H band (2489.5 cm^−1^) might not be observed apparently for GSH-passivated AgNCs, implying that AgNCs were covered with the thiol groups-existing DHLA and GSH by the formation of Ag-S bonds. Moreover, AgNCs prepared by using the common template of DHLA might display the selective response to Hg^2+^ ions by fluorescence quenching (Fig. 1A), as also confirmed elsewhere^7^. However, one can note from Fig. 1B that upon the GSH passivation the AgNCs could show the specific ion recognition changing from Hg^2+^ ions to Cu^2+^ ions. Importantly, the simultaneous fluorimetric and colorimetric assays for Cu^2+^ ions could thus be expected as demonstrated afterwards.

**Figure 2:**
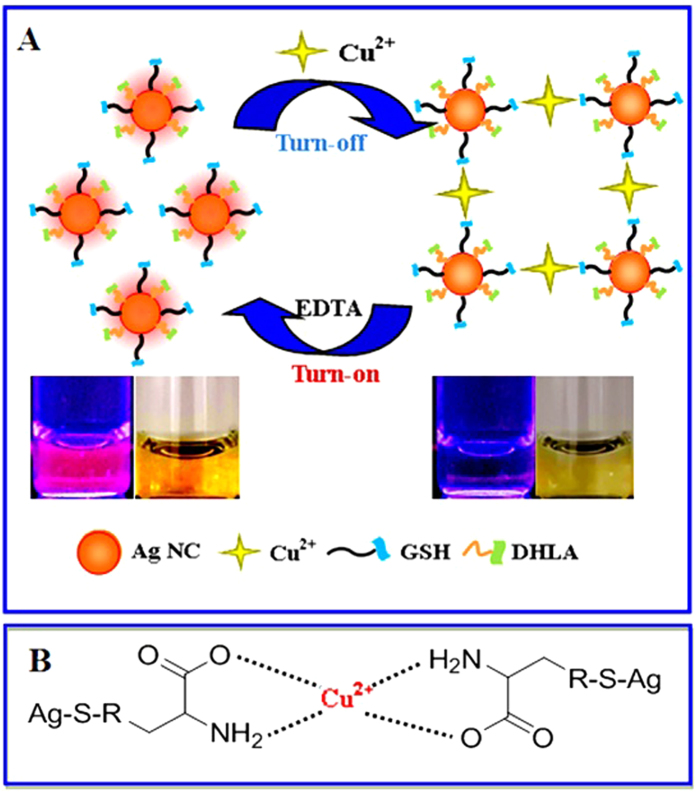
Schematic illustration of (**A**) the reversible fluorometric and colorimetric detection mechanism and procedure of GSH-passivated AgNCs toward the fluorimetric and colorimetric assays for Cu^2+^ ions (insert: the photographs of corresponding products), and (**B**) the chelating interaction between Cu^2+^ ions and GSH of GSH-passivated AgNCs.

**Figure 3:**
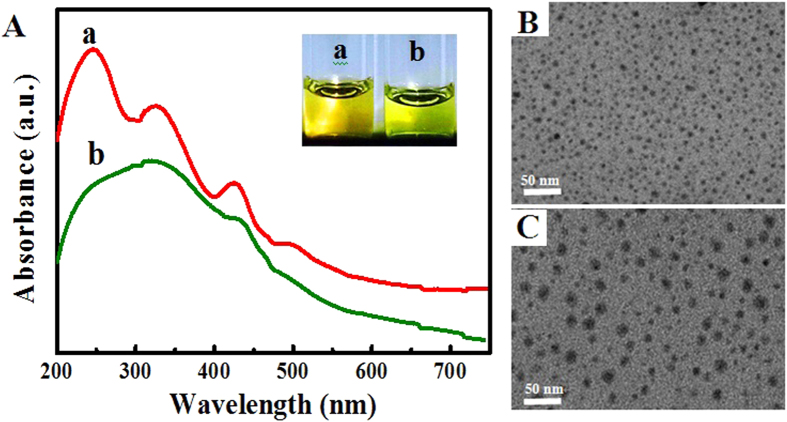
(**A**) UV-vis spectra of GSH-passivated AgNCs (1.0 μM) in the (a) absence and (b) presence of Cu^2+^ ions (0.50 μM) (insert: the photographs of the testing solutions), with (**B**,**C**) the corresponding TEM images, respectively.

The fluorometric and colorimetric detection mechanism and procedure for Cu^2+^ ions is schematically illustrated in Fig. 2A. The addition of Cu^2+^ ions could markedly quench (“turn off”) the red fluorescence of GSH-passivated AgNCs together with a yellow color change via the GSH-Cu^2+^ interaction. Herein, GSH of GSH-passivated AgNCs may chelate with Cu^2+^ ions in molar ratio 2:1 via its carboxyl and amine groups^22^,^23^,^24^, attaining the preferentially stable tetragonal geometry of Cu^2+^-GSH complex^25^, as schematically described in Fig. 2B. As a result, the Cu^2+^-induced specific aggregation of GSH-passivated Ag NCs could undergo resulting in the fluorescence quenching and a decrease in the UV-vis absorbance. It is worth pointing out that the fluorescence quenching of GSH-passivated AgNCs might be partly attributed to the energy transfer between GSH-passivated AgNCs and Cu^2+^ ions that possess the strong absorbance in the range of red light^8^. Moreover, the fluorescence lifetime of GSH-passivated AgNCs might not be significantly changed in the presence and absence of Cu^2+^ ions (data not shown). However, an obvious change in the UV-vis absorbance spectra could be observed for GSH-passivated AgNCs in the presence and absence of Cu^2+^ ions (Fig. 3A). These evidences suggested that Cu^2+^ ions might conduct the static fluorescence quenching of GSH-passivated AgNCs^26^. In addition, the lost fluorescence of the resultant GSH-passivated AgNCs could be restored by introducing a Cu^2+^ chelating agent, i.e., ethylenediaminetetraacetate (EDTA) with the considerably high Cu^2+^-binding affinity^21^, to release the GSH-bound Cu^2+^ ions from the surface of GSH-passivated AgNCs.

Figure S2 manifests the quantitative comparison of the fluorescence spectra and UV-vis spectra for the EDTA-enabled reversible responses of GSH-passivated AgNCs to Cu^2+^ ions. As expected, the lost fluorescence (Figure S2A) and UV-vis absorbance (Figure S2B) could be restored over 90% of their initial values by using EDTA. Of note, the emissive fluorescence peaks (650 nm) of the GSH-passivated AgNCs could display only a change in the fluorescence intensity after adding Cu^2+^ ions, so did the UV-vis absorbance peaks of AgNCs (330, 425, and 500 nm). In contrast, the UV-vis absorption peak (250 nm) of GSH of GSH-passivated AgNCs could show a red shift upon the addition of Cu^2+^ ions (Figure S2B). Again, the above phenomenon confirms that the Cu^2+^-induced optical change of GSH-passivated AgNCs should be attributed to the Cu^2+^-GSH binding. Moreover, the topological structures of the GSH-passivated AgNCs in the absence and presence of Cu^2+^ ions were characterized by transmission electron microscopy (TEM) imaging (Fig. 3B,C). It was observed that GSH-passivated AgNCs could be well dispersed in the aqueous media with the uniform particle size (Fig. 3B). Once Cu^2+^ ions (0.50 μM) were introduced, they could get aggregation with the greatly increased particle size (Fig. 3C), so that their average hydrodynamic diameter could change from about 4.3 nm to about 17.5 nm (Figure S3).

The aqueous stability of GSH-passivated AgNCs was investigated over different time intervals of storage under different ionic strengths in NaCl concentrations (Figure S4). Surprisingly, GSH-passivated AgNCs could retain well their fluorescence intensities up to 1.0 M NaCl (Figure S4A), which otherwise could act as a strong precipitate agent for silver ions, achieving the stable storage for six months (Figure S4B). Accordingly, GSH-passivated AgNCs could survive under the harsh conditions, thus promising the potential applications in some complicated media like blood. Furthermore, the pH-dependent fluorescent responses of GSH-passivated AgNCs to Cu^2+^ ions were examined (Figure S5A), showing the optimal response at about pH 7.0. In addition, a fast response (about one min) to Cu^2+^ ions could be obtained for GSH-passivated AgNCs (Figure S5B).

**Figure 4:**
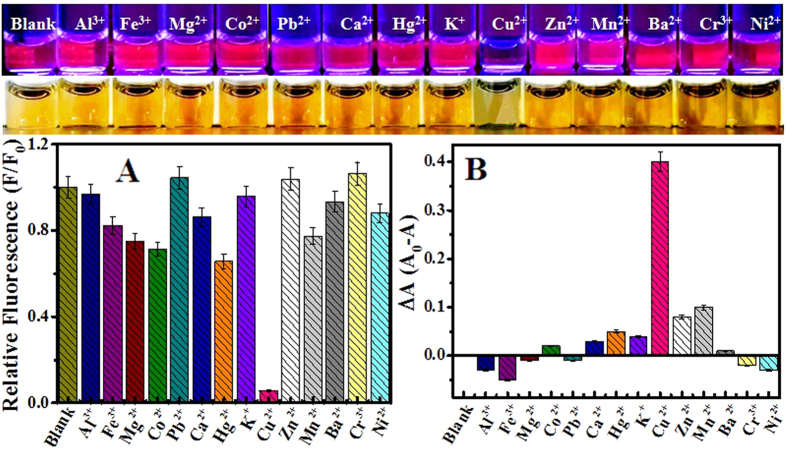
Comparable investigation of (**A**) fluorometric and (**B**) UV-vis colorimetric responses of GSH-passivated AgNCs (5.0 μM) to Cu^2+^ ions (1.0 μM) and other metal ions of Al^3+^, Fe^3+^, Mg^2+^, Co^2+^, Pb^2+^, Ca^2+^, Hg^2+^, K^+^, Cu^2+^, Zn^2+^, Mn^2+^, Ba^2+^, Cr^3+^, and Ni^2+^ ions (10 μM), with the corresponding photographs of the products under (top) UV and (bottom) visible light.

The specific fluorometric and colorimetric responses of GSH-passivated AgNCs to Cu^2+^ ions were further explored comparing to some common metal ions including Al^3+^, Fe^3+^, Mg^2+^, Co^2+^, Pb^2+^, Ca^2+^, Hg^2+^, K^+^, Zn^2+^, Mn^2+^, Ba^2+^, Cr^3+^, and Ni^2+^ ions (Fig. 4). Obviously, other metal ions including Hg^2+^ ions caused no significant change in the fluorescence and UV-vis absorbance of GSH-passivated AgNCs, even at the concentrations of 10-fold higher than that of Cu^2+^ ions. Furthermore, the investigation was conducted on the fluorescent responses of GSH-passivated AgNCs to Cu^2+^ ions in the mixtures separately co-existing other metal ions (Figure S6), showing the selective sensing of Cu^2+^ ions. Therefore, the introduction of GSH could effectively modulate the specific ion recognition of AgNCs from Hg^2+^ ions to Cu^2+^ ions, thus facilitating the selective fluorometric and colorimetric assays for Cu^2+^ ions.

**Figure 5:**
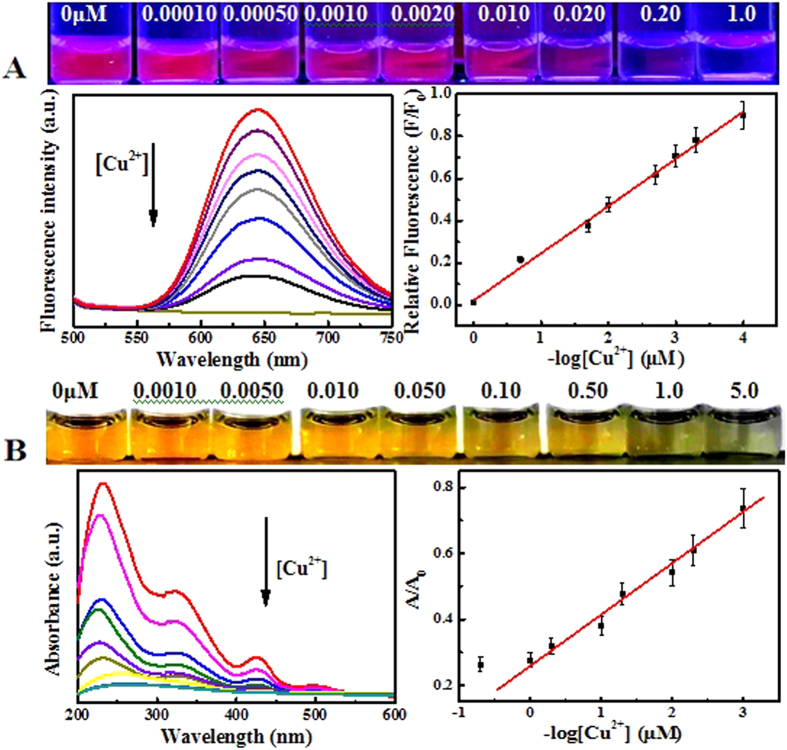
(**A**) Typical fluorescent emission spectra (left) and the calibration curve (right) of GSH-passivated AgNCs (5.0 μM) versus Cu^2+^ ions of different concentrations, with the corresponding photographs of testing solutions under UV light (top). (**B**) Typical UV-vis spectra (left) and the calibration curve (right) of GSH-passivated AgNCs (5.0 μM) versus Cu^2+^ ions of different concentrations, with the corresponding photographs of detection solutions under white light (top).

Under the optimized conditions, the fluorometric assays for Cu^2+^ ions with different concentrations were performed using GSH-passivated AgNCs as the probes (Fig. 5A). Accordingly, the fluorescence intensities of GSH-passivated AgNCs could decrease with increasing Cu^2+^ concentrations. A linear detection range could be achieved for Cu^2+^ concentrations ranging from 0.00010 to 1.0 μM, with the limit of detection (LOD) of about 0.050 nM, estimated by the 3σ rule. Moreover, the colorimetric analysis of Cu^2+^ ions was carried out (Fig. 5B). The UV-vis spectra of GSH-passivated AgNCs could change depending on the Cu^2+^ concentrations. A calibration detection curve was also obtained over the linear concentration range of Cu^2+^ ions of 0.0010–1.0 μM, with the LOD of about 0.60 nM. In addition, a comparison of LOD for Cu^2+^ ions was conducted among the GSH-passivated AgNCs-based fluorimetry and those analysis methods reported previously, indicating the developed assay could present the lower LOD (Table S1). In addition, the feasibility of the practical applications of the fluorometic and colorimetric assays were explored for Cu^2+^ ions spiked in blood with different concentrations (Figure S7), showing the LOD of 0.10 nM and 0.80 nM, respectively. Therefore, the developed analysis strategy could facilitate the simultaneous fluorometric and colorimetric assays for Cu^2+^ ions in blood with high detection sensitivity.

**Figure 6:**
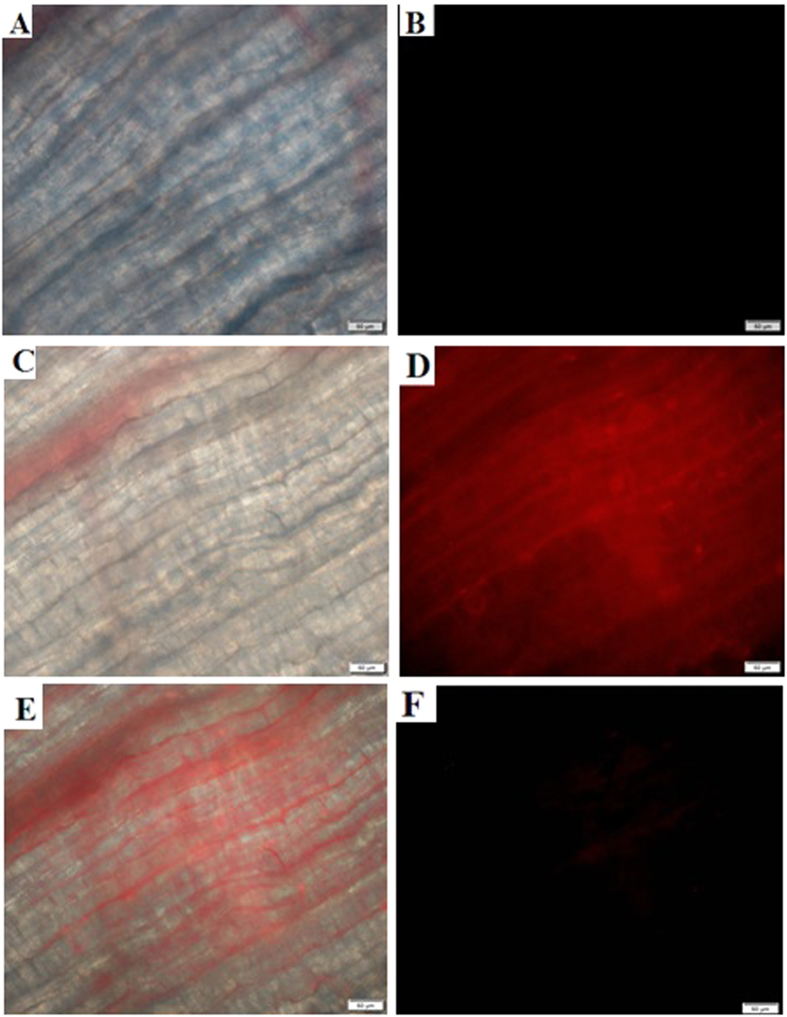
Microscopy fluorescent images of live mouse muscle tissues before (**A,B**) and after (**C,D**) being treated with GSH-passivated AgNCs (20 μM) in the bright field (**A,C**) and dark field (**B,D,E**) the overlay image of (**C,D,F**) the image of live mouse muscle tissues treated with GSH-passivated AgNCs (20 μM) containing Cu^2+^ ions (10 μM) in dark field. The measurement conditions were detailed in the Experimental.

Moreover, cytotoxicity (uptake) studies *in vitro* level were conducted on GSH-passivated AgNCs for culturing yeast cells, with the data shown in Figure S8. The results showed that GSH-passivated AgNCs could present no significant toxicity to the live cells even at a high concentration. Furthermore, the feasibility of fluorescence imaging for yeast cells was also demonstrated in the absence and presence of Cu^2+^ ions, with the images shown in Figure S9. One can note that the GSH-passivated AgNCs with red fluorescence could light up the yeast cells, of which the fluorescence might be turned off after Cu^2+^ ions were introduced into the media of cell culture. Furthermore, GSH-passivated AgNCs were employed for the fluorescence-trackable imaging for the live muscle tissues separately from the back and leg parts of a mouse in narcosis, with the typical images shown in Fig. 6. It was observed clearly from the dark-field images that the muscle tissues, which might present no fluorescence (Fig. 6B), could display strong red fluorescence after being treated with GSH-passivated AgNCs (Fig. 6D). In contrast, the injection of Cu^2+^ ions into the live mouse muscle tissues pretreated with GSH-passivated AgNCs could gradually fade out their red fluorescence (Fig. 6F). These evidences indicated that the GSH-passivated AgNCs, which possess the powerful red fluorescence, good biocompatibility, and high aqueous stability, could be tailored for the fluorescence-trackable imaging of live cells and tissues towards the analysis of Cu^2+^ ions. The extensive biological applications for trackable biotoxicity study and drug delivery in the complicated media like blood could also be expected.

In summary, AgNCs were successfully synthesized and passivated by GSH ligands, so that the specific ion recognition was modulated from Hg^2+^ ions to Cu^2+^ ions that could trigger the aggregation of nanoclusters toward the fluorescence quenching. The obtained GSH-passivated AgNCs could display the powerful red fluorescence and high aqueous stability. Meantime, a rational change of UV-vis yellow absorbance spectra of GSH-passivated AgNCs could be obtained depending on the Cu^2+^ levels. Moreover, the Cu^2+^-induced loss of fluorescence and UV-vis absorbance of GSH-passivated AgNCs could be efficiently restored by using the Cu^2+^ chelating agent of EDTA. A simultaneous and reversible fluorometric and colorimetric analysis protocol using GSH-passivated AgNCs has thereby been proposed for probing Cu^2+^ ions with high detection sensitivity and selectivity. Also, the application feasibility of fluorescence-trackable imaging for live cells and tissues was verified using GSH-passivated AgNCs as the fluorescent probes. The results indicate that GSH-passivated AgNCs could not only facilitate the selective analysis of the Cu^2+^ ions in complicated media, but also allow for the biological imaging for live cells and tissues, where the background interferences from the endogenous fluorescent species could be efficiently minimized. Such a ligand passivation route may open a new door for modulating the specific ion recognition of noble metal NCs toward the huge applications of the useful optical probes in the functional material synthesis, ion sensing, biological imaging, and ion-adsorption separation of biological and chemical interests.

## Methods

### Materials and Instruments

Glutathione (GSH), α-lipoic acid (LA), silver nitrate (AgNO_3_), sodium hydroxide, sodium borohydride, copper nitrate (Cu (NO_3_)_2_), sodium chloride, ethylenediaminetetraacetate (EDTA), yeast cells, thiazolyl blue tetrazolium bromide (MTT) were purchased from Sigma-Aldrich (Beijing, China). Yeast peptone dextrose agar (YPD) was obtained from Aladdin. The blood samples were kind provided by the local hospital. All of the chemicals were of analytical grade, and all glass containers were cleaned by aqua regia and ultrapure water.

Fluorescence spectrophotometer (F-7000, Hitachi, Japan), UV-3600 spectrophotometer (Shimadzu, Japan), Fourier transform infrared spectrophotometer (FT-IR, Thermo Nicolet Nexus 470FT, USA) and Transmission electron microscopy (TEM, Tecnai G20, FEI, USA) operated at 100 kV were employed to characterize the products of AgNCs in the absence and presence of Cu^2+^ ions. The hydrodynamic diameters of AgNCs before and after Cu^2+^ treatment were measured comparably by dynamic light scattering (DLS) with a Zetasizer Nano ZS (Malvern Instruments, UK) setup equipped with a helium-neon laser (λ = 632.8 nm, 4.0 mW). Tissue and cell imaging was observed using the fluorescent inverted microscope (Olympus, IX73-DP80, Japan).

### Synthesis of GSH-passivated Ag nanoclusters

Typically, the synthesis of GSH-passivated AgNCs was conducted by adding 125 μL AgNO_3_ (20 mM) and 150 μL GSH (50 mM) into 5.0 mL ultrapure water at room temperature by magnetic stirring. Then, NaOH (1.0 M) was used to adjust pH to 9.0. At the same time, 5.0 mg LA was mixed with pure sodium borohydride in the molar ratio of LA:NaBH_4_ = 4:1 and this was stirred well until a clear solution was observed. Here, LA was first reduced in water to form soluble dihydrolipoic acid (DHLA). Then, the prepared DHLA solution was immediately added into the silver mixture by vigorously stirring for one min. Subsequently, a slightly excessive sodium borohydride solution was added dropwise into the mixture. After stirring for 20 min, the mixture was further incubated for 1.5 h at room temperature. After the dialysis and purification, the resulting GSH-passivated AgNCs were stored at 4 °C in the fridge. In addition, the general AgNCs were synthesized according to the procedure reported previously^7^.

### Fluorimetric and colorimetric analysis

Fluorimetric and colorimetric assays with GSH-passivated AgNCs for Cu^2+^ ions were conducted by the following steps. Typically, an aliquot of GSH-passivated AgNCs were dispersed in the buffer (pH 7.0). Then, a desirable amount of Cu^2+^ ions with different concentrations was added to be mixed and further incubated for 5 min. Subsequently, the fluorimetric and colorimetric measurements were performed to record the changes of the fluorescence intensities and UV absorbances of the sensing reactions. Moreover, the experiments for the restoring fluorescence of GSH-passivated AgNCs were carried out by using GSH-passivated AgNCs (5.0 μM), Cu^2+^ ions (0.80 μM), and EDTA (1.6 μM). In addition, the fluorimetic and colorimetric detections were conducted accordingly for Cu^2+^ ions spiked in blood samples with different concentrations.

### Fluorescence-trackable imaging of live cells and tissues

The cytotoxicity tests were firstly performed. An aliquot of yeast cells (1 × 10^5^) in 50 μL buffer was seeded into each of the testing wells on a 96-well plate. After the culture overnight, 50 μL of GSH-passivated AgNCs (0–14.0 μM) or GSH-passivated AgNCs containing Cu^2+^ ions were separately introduced into the testing wells to be treated for 2 h. Then, an aliquot of 10 μL of MTT solution was added into each of the wells, followed by the incubation at 37 °C for 4 h. Furthermore, an aliquot of 100 μL of solubilization solution containing 10% SDS and 0.010 M HCl was separately added to dissolve the purple crystals by incubating for 12 h. Subsequently, the optical density readings were taken at 595 nm to record the viabilities of yeast cells so cultured using a plate reader.

The fluorescence-trackable imaging was performed for yeast cells. The live cells were firstly centrifugated at 8000 rpm for 5.0 min, and then washed with 0.85% NaCl. Following that, the cell samples were diluted and spread on the YPD agar plates to be incubated overnight at 37 °C. After washing for several times, an aliquot of GSH-passivated AgNCs (10 μM) was injected and incubated at 37 °C for 1 h, followed by washing twice. The resulting cell mixtures were separately dropped onto the slides to be imaged separately in the light and dark fields using the fluorescent inverted microscope. Furthermore, an aliquot of Cu^2+^ ions (5.0 μM) was injected to the mixtures to be incubated at 37 °C for 1 h. Subsequently, the fluorescence imaging of the resulting samples of yeast cells was conducted accordingly.

Moreover, the fluorescence-trackable imaging was carried out for the live muscle tissues separately from the back and leg parts of a mouse in narcosis. An aliquot of GSH-passivated AgNCs (20 μM) was separately injected into the muscle tissues to be treated for 30 min. Then, the fluorescent imaging was conducted for the resulting muscle tissues spread on the slides, taking the ones without GSH-passivated AgNCs as the controls. Furthermore, an aliquot of Cu^2+^ ions (10 μM) were injected into the live muscle tissues pre-treated with GSH-passivated AgNCs. After being treated further for 1 h, the fluorescent imaging was finally conducted according to the same procedure above.

## Additional Information

**How to cite this article**: Sun, Z. *et al*. Silver Nanoclusters with Specific Ion Recognition Modulated by Ligand Passivation toward Fluorimetric and Colorimetric Copper Analysis and Biological Imaging. *Sci. Rep.* **6**, 20553; doi: 10.1038/srep20553 (2016).

## Supplementary Material

Supplementary Information
