# Supplementary material for: Silver Nanoclusters with Specific Ion Recognition Modulated by Ligand Passivation toward Fluorimetric and Colorimetric Copper Analysis and Biological Imaging
Source: Sci Rep. 2016 Feb 5;6:20553. doi: 10.1038/srep20553 (PMC4742814; doi:10.1038/srep20553)
Supplement: Supplementary Information [file srep20553-s1.doc]

**Supplementary Information for**

**Silver Nanoclusters with Specific Ion Recognition Modulated by Ligand Passivation toward Fluorimetric and Colorimetric Copper Analysis and Biological Imaging**

Zongzhao Sun,^1^ Shuying Li,^1^ Yao Jiang,^1^ Yuchun Qiao,^1^ Liyan Zhang,^1^ Lulu Xu,^1^ Jinghui Liu,^1^ Wei Qi,^1^ & Hua Wang^1,2^*

^1^ Shandong Province Key Laboratory of Life-Organic Analysis, College of Chemistry and Chemical Engineering, Qufu Normal University, Qufu, 273165, P. R. China.

^2^ Jining Functional Materials and Surface Treatment Technology R & D Center, Southern Shandong Academy of Engineering Technology, Jining City, Shandong Province 272000, P. R. China.

* E-mail: huawangqfnu@126.com; Tel: (+86) 5374456306; Web: http://wang.qfnu.edu.cn.

.

**List of Contents**

**Table S1.** Comparison of the limit of detections (LODs) among different analysis methods for Cu^2+^ ions.

**F****igure** **S1**. FT-IR spectra of (a) GSH, (b) DHLA, and (c) GSH-passivated AgNCs.

**Figure S2.** (A) Comparison of fluorescence spectra at λ_em_ 650 nm and (B) UV-vis spectra of GSH-passivated AgNCs (5.0 μM) in the (a) absence and (b) presence of EDTA, (c) Cu^2+^ ions, and (d) Cu^2+^ ions with EDTA, where 0.80 μM Cu^2+^ ions and 1.6 μM EDTA were used.

**Figure S3.** Hydrodynamic diameters of GSH-passivated AgNCs (5.0 μM) in the (A) absence and (B) presence of Cu^2+^ ions (0.50 μM) by DLS measurements.

**Figure S4.** Stability investigation of GSH-passivated AgNCs stored (A) in the different ionic strengths in NaCl concentrations and (B) in water over the different time intervals at 4 ^o^C.

**Figure S5.** (A) The pH-dependent and (B) time-dependent fluorescence responses of GSH-passivated AgNCs to Cu^2+^ ions by fluorescence quenching in the (a) presence and (b) absence of Cu^2+^ ions (1.0 μM).

**Figure S6.** Fluorescence responses of GSH-passivated AgNCs (5.0 μM) to Cu^2+^ ions (0.010 μM) separately co-existing other metal ions (0.10 μM) including Al^3+^, Fe^3+^, Mg^2+^, Co^2+^, Pb^2+^, Ca^2+^, Hg^2+^, K^+^, Zn^2+^, Mn^2+^, Ba^2+^, Cr^3+^, and Ni^2+^ ions.

**Figure S7.** Calibration detection curves for (A) fluorimetric analysis with relative florescence intensities vs. different concentrations of Cu^2+^ ions from 0.00050 μM to 0.80 μM in blood, and (B) colorimetric analysis with UV-vis absorbance values vs. Cu^2+^ ions spiked in blood with different concentrations from 0.0010 μM to 1.0 μM.

**Figure S8.** Viabilities of yeast cells cultured in the presence of (A) GSH-passivated AgNCs and (B) GSH-passivated AgNCs with Cu^2+^ ions, of which the MTT assays were conducted separately for the yeast cells with different concentrations of GSH-passivated AgNCs (0 - 14.0 μM) and GSH-passivated AgNCs (10 μM) containing different concentrations of Cu^2+^ ions (2.0 - 10.0 μM).

**Figure S9.** Fluorescence imaging of yeast cells after being treated with GSH-passivated AgNCs (10 μM) in the (A) absence and (B) presence of Cu^2+^ ions (5.0 μM).

**Table S1.** Comparison of the limit of detections (LODs) among different analysis methods for Cu^2+^ ions.

| **Detection methods** | **Probes** | **LODs** | **References** |
| --- | --- | --- | --- |
| Colorimetric assay | GSH-passivated AgNCs | 0.60 nM | This work |
|  | Au nanorods | 1.6 nM | Ref.[1](#_ENREF_1) |
| Fluorimetric assay | GSH-passivated AgNCs | 0.050 nM | This work |
|  | BSA-Au NCs | 1.0 nM | Ref. [2](#_ENREF_2) |
| Electrochemical assay | Ag NPs | 0.48 nM | Ref. [3](#_ENREF_3) |

1. Niu, X.; Xu, D.; Yang, Y. & He, Y. Ultrasensitive colorimetric detection of Cu^2+^ using gold nanorods. Analyst. **139**, 2691-2694 (2014).

2. Liu, H.; Zhang, X.; Wu, X.; Jiang, L.; Burda, C. & Zhu, J. J. Rapid sonochemical synthesis of highly luminescent non-toxic AuNCs and Au@ AgNCs and Cu (II) sensing. Chem. Commun.. **47**, 4237-4239 (2011).

3. Cui, L.; Wu, J.; Li, J.; Ge, Y. & Ju, H. Electrochemical detection of Cu< sup> 2+</sup> through Ag nanoparticle assembly regulated by copper-catalyzed oxidation of cysteamine. Biosens. Bioelectron.. **55**, 272-277 (2014).

**Figure** **S1**. FT-IR spectra of (a) GSH, (b) DHLA, and (c) GSH-passivated AgNCs.

**Figure S2.** (A) Comparison of fluorescence spectra at λ_em_ 650 nm and (B) UV-vis spectra of GSH-passivated AgNCs (5.0 μM) in the (a) absence and (b) presence of EDTA, (c) Cu^2+^ ions, and (d) Cu^2+^ ions with EDTA, where 0.80 μM Cu^2+^ ions and 1.6 μM EDTA were used.


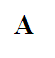

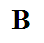


**Figure S3.** Hydrodynamic diameters of GSH-passivated AgNCs (5.0 μM) in the (A) absence and (B) presence of Cu^2+^ ions (0.50 μM) by DLS measurements.


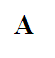

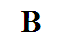


**Figure S4.** Stability investigation of GSH-passivated AgNCs stored (A) in the different ionic strengths in NaCl concentrations and (B) in water over the different time intervals at 4 ^o^C.


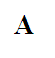

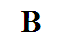


**Figure S5.** (A) The pH-dependent and (B) time-dependent fluorescence responses of GSH-passivated AgNCs to Cu^2+^ ions by fluorescence quenching in the (a) presence and (b) absence of Cu^2+^ ions (1.0 μM).

**Figure S6.** Fluorescence responses of GSH-passivated AgNCs (5.0 μM) to Cu^2+^ ions (0.010 μM) separately co-existing other metal ions (0.10 μM) including Al^3+^, Fe^3+^, Mg^2+^, Co^2+^, Pb^2+^, Ca^2+^, Hg^2+^, K^+^, Zn^2+^, Mn^2+^, Ba^2+^, Cr^3+^, and Ni^2+^ ions.


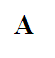

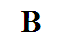


**Figure S7.** Calibration detection curves for (A) fluorimetric analysis with relative florescence intensities vs. different concentrations of Cu^2+^ ions from 0.00050 μM to 0.80 μM in blood, and (B) colorimetric analysis with UV-vis absorbance values vs. Cu^2+^ ions spiked in blood with different concentrations from 0.0010 μM to 1.0 μM.


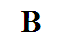

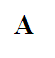


**Figure S8.** Viabilities of yeast cells cultured in the presence of (A) GSH-passivated AgNCs and (B) GSH-passivated AgNCs with Cu^2+^ ions, of which the MTT assays were conducted separately for the yeast cells with different concentrations of GSH-passivated AgNCs (0 - 14.0 μM) and GSH-passivated AgNCs (10 μM) containing different concentrations of Cu^2+^ ions (2.0 - 10.0 μM).


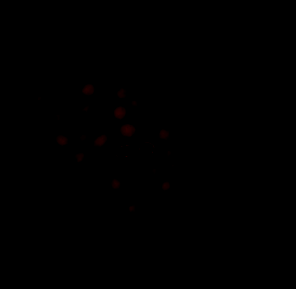

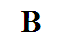

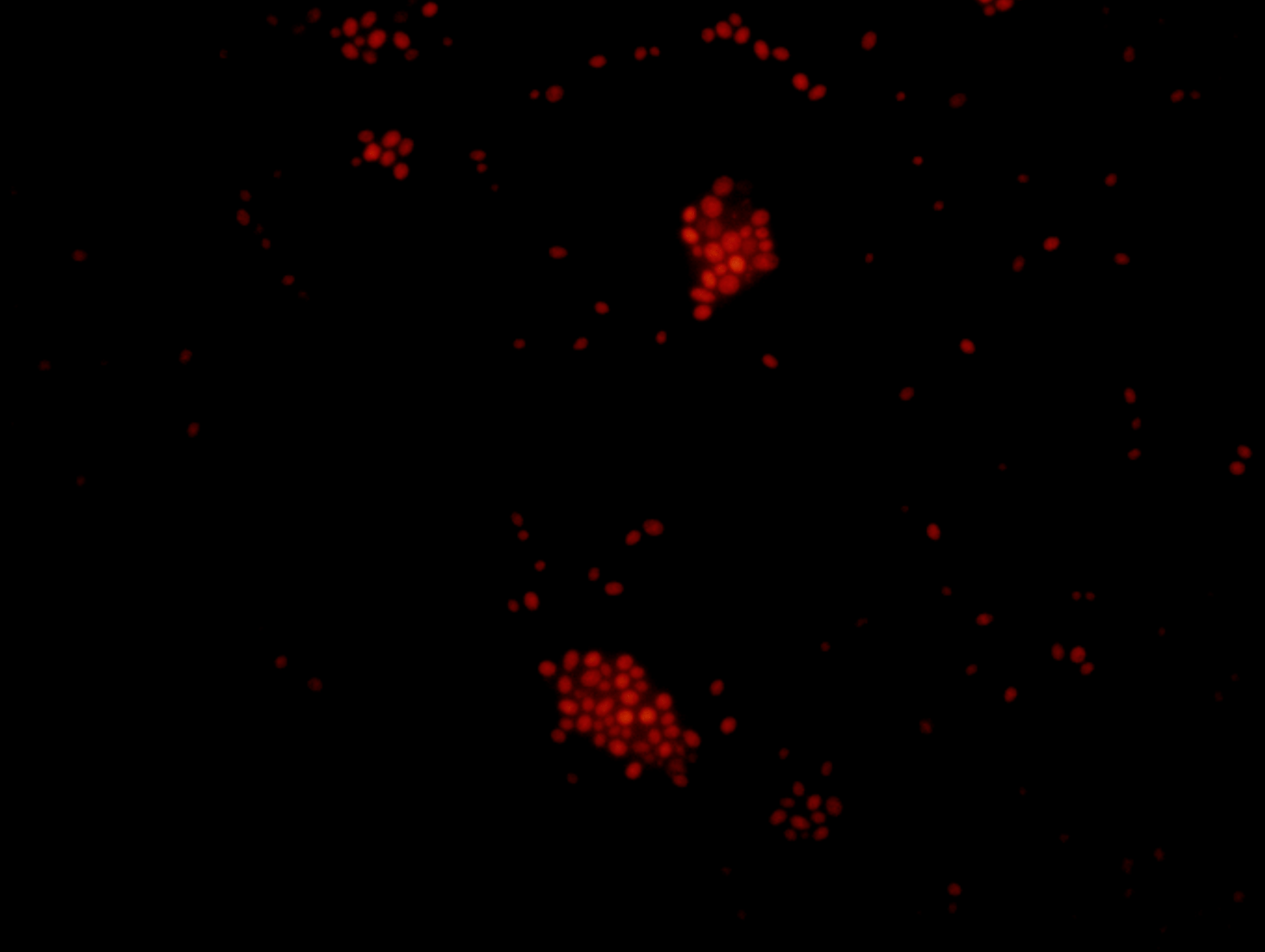

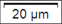

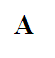


**Figure S9.** Fluorescence imaging of yeast cells after being treated with GSH-passivated AgNCs (10 μM) in the (A) absence and (B) presence of Cu^2+^ ions (5.0 μM).
